# Incidence and Risks of Congenital Anomalies of Kidney and Urinary Tract in Newborns: A Population-Based Case–Control Study in Taiwan: Erratum

**DOI:** 10.1097/01.md.0000482789.12887.33

**Published:** 2016-04-18

In the article “Incidence and Risks of Congenital Anomalies of Kidney and Urinary Tract in Newborns: A Population-Based Case–Control Study in Taiwan”,^1^ which appeared in Volume 95, Issue 5 of *Medicine*, a grant number was originally given incorrectly. The article has since been corrected online.
